# Disentangling Litter Size and Farrowing Duration Effects on Piglet Stillbirth, Acid–Base Blood Parameters and Pre-Weaning Mortality

**DOI:** 10.3389/fvets.2022.836202

**Published:** 2022-04-21

**Authors:** Moniek van den Bosch, Irene B. van de Linde, Bas Kemp, Henry van den Brand

**Affiliations:** ^1^Cargill Animal Nutrition Innovation Center Velddriel, Cargill b.v., Velddriel, Netherlands; ^2^Adaptation Physiology Group, Wageningen University and Research, Wageningen, Netherlands

**Keywords:** farrowing duration, litter size, stillbirth, pre-weaning mortality, piglets, acid–base blood parameters

## Abstract

The current study evaluated interactions between farrowing duration and litter size on the level of asphyxia, vitality, percentage of stillbirth, and pre-weaning mortality of piglets. Farrowing duration was measured in 159 crossbred gilts and sows (Yorkshire × Dutch Landrace). Litter size ranged between 12 and 21 piglets. Blood acid–base parameters in umbilical cord blood and vitality scores of piglets were determined immediately after birth. Number of piglets born alive and stillborn as well as individual piglet weights at birth were recorded. Pre-weaning mortality (excluding stillbirth) was determined throughout lactation. Litter size as well as farrowing duration were categorized to evaluate the interaction between the two. There tended to be an interaction between litter size and farrowing duration for pre-weaning mortality (*p* = 0.10). In small litters (12–15 piglets), a prolonged farrowing duration (>250 min) tended to increase pre-weaning mortality compared with a short (<150 min) and medium farrowing duration (150–250 min), while for large litters (19–21 piglets), a medium to long farrowing duration tended to decrease pre-weaning mortality. No other interactions between litter size and farrowing duration were found. Piglets within large litters showed a higher umbilical cord lactate level (*p* < 0.01), lower average vitality score (*p* = 0.01), and a higher stillborn percentage (*p* < 0.01) compared with piglets within medium size (16–18 piglets) and small litters. Each additional piglet born to a litter linearly decreased average piglet birth weight (17.6 g, *p* < 0.01), increased farrowing duration (11 min, *p* < 0.01), and increased stillbirth (0.5%, *p* = 0.04). A medium farrowing duration resulted in a lower stillborn percentage compared with a short or prolonged farrowing duration, suggesting that farrowing duration might have an optimum. When analyzed linearly, stillborn percentage increased with 1.85% per every 100 min (*p* < 0.01) of farrowing duration. It can be concluded that both litter size and farrowing duration affect stillborn percentage, but independent from each other. However, these two factors tended to interact regarding pre-weaning mortality, suggesting that setting a certain threshold for maximal farrowing duration should be taken with care, because this appears to depend on litter size.

## Introduction

Larger litter sizes in pigs are often accompanied by a higher incidence of stillbirth (1–4) with a prolonged farrowing duration being a key driver for this (5). A longer farrowing duration has been associated with a higher risk of hypoxia in piglets (6), due to succesive uterine contractions, reduction of utero-placental blood flow (7), or loss of the umbilical cord functionality (8, 9). Depending on the severity, hypoxia may lead to stillbirth or a lower piglet vitality, potentially leading to pre-weaning mortality (10). Several studies have suggested that farrowing duration should not exceed a certain threshold level. For example, Oliviero et al. (11) suggested that farrowing duration should not take longer than 300 min, since incidence of stillbirth increased from 0.4 to 1.5 stillborn piglets in litters with a farrowing duration below or above the threshold level of 300 min. Langendijk et al. (12) showed that stillbirth incidence increased exponentially when farrowing duration took more than 240 min. Stillbirth incidence was 2.7, 6.9, 10.7, 13.4, and 27.3% when farrowing duration was <120, 120–240, 240–360, 360–480, and more than 480 min, respectively. They suggested to intervene in the farrowing process from a farrowing duration of 240 min onward. Interestingly, the average litter size in the studies of Oliviero et al. (11) and Langendijk et al. (12) differed considerably (12.7 ± 3.0 and 15.3 ± 0.5 piglets born in total, respectively), whereas the suggested threshold level showed the opposite effect. This suggests that other factors (e.g., breed, sow body condition, average parity of the herd, stress, and also environmental factors like supervised farrowing, the use hormones, climate, etc.) can affect the threshold level above which farrowing duration might have negative effects on piglet suvival rates. One of the interfering factors might be litter size. Farrowing duration is positively related to litter size (13), which might suggest that optimal duration of farrowing depends on litter size. Consequently, it can be speculated that for medium (≥16 total born) or large litters (≥19 total born), prolonged farrowing duration is less detrimental than for smaller litters (12–15 piglets total born), since it simply takes more time to farrow more piglets. The aim of this research was to disentangle effects of litter size at birth and farrowing duration on the level of hypoxia and incidence of stillbirth and pre-weaning mortality.

## Materials and Methods

### Animals

In total, 190 gilts and sows (Yorkshire × Dutch Landrace, Topigs Norsvin) of parity 1 to 9, in eight consecutive batches were used in this study. The study was performed at the Swine Innovation Center Sterksel of Wageningen University and Research, The Netherlands. Per batch, four farrowing units were used, each containing 12 farrowing pens. Animals entered the farrowing room ~7 days before the expected farrowing date (i.e., day 115 after insemination) and were placed in individual farrowing crates (pen size 180 × 240 cm, crate size of 55 × 185 cm). No nesting material was provided. Data of sows used in the current study were obtained during a feeding experiment, evaluating effects of supplementing 0.00, 0.03, 0.06, 0.09, 0.12, and 0.15% of nitrate in the perinatal period on piglet survival as described by Van den Bosch et al. (14, 15). Lactation diets were provided twice daily (0730 H and 1,630 H) from the moment sows entered the farrowing room (day 108 ± 1 of gestation) until weaning (day 27.2 ± 1.7 postpartum). Sows had *ad libitum* access to water. Each pen had a piglet nest with a heating lamp set at 30°C. During farrowing, supervision was present for 24 h a day, but it was not allowed to use any intervention during farrowing or interfere with piglet survival after birth by, e.g., saving them from crushing or placing them at the udder or in the piglet nest. Farrowing was not induced, and sows that received birth assistance or medication during farrowing were excluded from the experiment. Cross-fostering took place between sows that farrowed on the same day and only between 24 and 48 h after birth. Litters were standardized aiming for 15 piglets per sow. Piglets that were fostered on or off the sow were selected randomly. The number of dead piglets, reason for death (e.g., crushing, splay legs, starvation, lameness, weak, low birth weight, and unknown as scored by the farm staff), and weight of dead piglets were registered on a daily basis. Pre-weaning mortality excluded stillborn piglets. Pre-weaning mortality was calculated by the following equation:


$$\begin{array}{l} \text{Pre }-\text{ weaning mortality }=\left(\frac{\text{Number of pre }-\text{ weaning deaths }(\text{excluding TSB})}{\text{TBA }+\text{ number of piglets added }-\text{ number of piglets removed at cross fostering}}\right)\;\times \;100\% \end{array}$$

Piglets received a commercially available pre-starter from 3 days of age until weaning (17.4% CP, 11.6 MJ NE/kg; Top Wean, Agrifirm, Apeldoorn, the Netherlands).

### Measurements

Within 3 min after birth, a mixed blood sample (i.e., from the vein and/or artery) was taken from the umbilical cord (intact or ruptured) from two randomly chosen live-born piglets out of every four subsequent piglets born. A 2.5-ml syringe with a 16-mm 21 G needle and sodium oxalate as an anticoagulant was used. Blood acid–base parameters were analyzed within 1 min after collection, using the iStat® portable Clinical Analyzer (iStat Europe, Birmingham, UK) and CG8^+^ cartridges. Piglets were not handled during sampling to prevent stress. After blood sampling, piglets were tagged to record their birth order. The remaining blood was collected in a 4-ml BD vacutainer® (fluor heparin tube) and stored on ice before being centrifuged at 3,000 rpm at 4°C for 10 min. Blood plasma was decanted and stored at −20°C for further analysis. Lactate concentration in plasma was determined, using an enzymatic UV test with lactate dehydrogenase with reagents of DiaSys Diagnostic Systems GmbH (Holzheim, Germany).

A digital video recorder (Samsung SRD470DP) connected to cameras (Velleman-CCD color cameras, Gavere, Belgium) was used to record the farrowing process of individual sows. Video recordings were analyzed using the Observer XT 10 software package (Noldus Information Technology B.V., Wageningen, the Netherlands). Video material of six sows could not be analyzed due to limited visibility. Individual vitality of newborn piglets was scored from video during the first 30 s after birth, using the scoring method as described by Baxter et al. (16):

0 = No movement, no breathing.

1 = No movement, but piglet is breathing or trying to breath (coughing and spluttering).

2 = Piglet is moving and breathing or trying to breath.

3 = Piglet is moving and breathing well and makes a first attempt to stand.

The total number of piglets born (excluding mummified and degenerating piglets), number of piglets born alive, and number of stillborn piglets were recorded after farrowing was completed. A stillborn piglet was defined as a piglet born without any respiration, but potentially with a heartbeat. A flotation test was performed to determine whether a piglet was a true stillborn. Approximately 2 cm^2^ of lung tissue was removed after dissection on the day of birth and placed in a bowl of water (17). When the lung tissue floated, the piglet was scored as a pre-weaning death instead of a stillborn. Both alive and stillborn piglets were individually weighed and numbered within 24 h after birth. Pre-weaning piglet mortality and weight of dead piglets were registered on a daily basis.

### Statistical Analyses

In total, the data of six sows were removed from the dataset due to receiving birth assistance (*n* = 3), sickness or death of the sow (*n* = 2), or receiving treatment for aggressive behavior during farrowing (*n* = 1). Sows that had a total litter size of <12 piglets (*n* = 11) or more than 21 piglets (*n* = 14) were excluded from the analyses due to limited number of sows per litter size. The final dataset contained the data of 159 litters. Residual plots were used to check model assumption (e.g., normality and equal variance of the error terms). Total duration of farrowing, which was defined as the time between the birth of the first and the last piglet in a litter, was non-normally distributed, and data were transformed by using a base 10 logarithm. Total stillborn piglets (TSBs) were found to be non-normally distributed even after transformation and were expressed as a percentage of total number born (TNB). Pre-weaning mortality was analyzed as a probability of the total number born alive (TBA).

To disentangle effects of litter size and farrowing duration or to test whether or not litter size and farrowing duration interact on piglet characteristics, litter size and farrowing duration were both categorized into three classes. Litter sizes of 12–15 piglets were classified as class 1 (*n* = 41), 16–18 piglets as class 2 (*n* = 71), and 19–21 piglets as class 3 (*n* = 47). Duration of farrowing was classified as <150 min (short; *n* = 59), 150–250 min (medium; *n* = 57), and over 250 min (long; *n* = 43). The GLIMMIX procedure in SAS (version 9.3, 2011, SAS Institute Inc., Cary, NC, USA) was used with the following model:


(1) $$\begin{array}{l} Y_{ijklmno}=\mu +\alpha_{i}+\beta_{j}+\alpha \beta_{ij}+c_{k}+d_{l}+f_{m}+g_{n}+\varepsilon_{ijklmno} \end{array}$$

where Y_*ijklmno*_ = dependent variable, μ = overall mean, α_*i*_ = fixed effect of litter size class (*i* = 1, 2, or 3), β_*j*_ = fixed effect of farrowing duration class (*j* = 1, 2, or 3), αβ_*ij*_ = the interaction between litter size class and farrowing duration class, c_*k*_ = random parity class effect (*k* = 1, 2, or 3; parity 1: class 1, parity 2, 3, and 4: class 2, and parity >4: class 3), d_*l*_ = random farrowing unit effect (*l* = 1, 2, …, 4), f_*m*_ = random feeding treatment effect (*m* = 1, 2, …,6), g_*n*_ = random batch effect (*n* = 1, 2, …, 8), and ε_*ijklmno*_ = residual error term. The sow was considered as the experimental unit.

Besides the categorized effect, also the fixed (reported as P_LS_) and linear effects of litter size (reported as P_LS Lin_) were assesed. Variables were subjected to the following statistical model, using a PROC GLIMMIX:


(2) $$\begin{array}{l} Y_{ijklmn}=\mu +\alpha_{i}+\beta_{j}+\alpha_{i}\beta_{j}+c_{k}+d_{l}+f_{m}+\varepsilon_{ijklmn} \end{array}$$

where Y_*ijklmn*_ = dependent variable, μ = overall mean, α_*i*_ = fixed effect of litter size (*i* = 12, 13, …, 21), β_*j*_ = fixed effect of farrowing duration, α_*i*_β_*j*_ = interaction between litter size and farrowing duration, c_*k*_ = random batch effect (*k* = 1, 2, …, 8), d_*l*_ = random parity class effect (*l* = 1, 2, or 3; parity 1: class 1, parity 2, 3, and 4: class 2, and parity >4: class 3), *f*_*m*_ = random feeding treatment effect (*m* = 1, 2, …, 6), and ε_*ijklmn*_ = residual error term. The sow was considered as the experimental unit. Additionally, the linear effect of litter size with other variables were assessed by using contrasts. For vitality score, the observer was added to the model as a random effect (*n* = 1, 2, or 3).

To access linear effects of farrowing duration (reported as P_FD_), variables were subjected to the following statistical model, using a PROC GLIMMIX:


(3) $$\begin{array}{l} Y_{ijklmn}=\mu +\alpha_{i}+\beta_{j}+\alpha_{i}\beta_{j}+c_{k}+d_{l}f_{m}+\varepsilon_{ijklmn} \end{array}$$

where Y_*ijklmn*_ = dependent variable, μ = overall mean, α_*i*_ = linear effect of farrowing duration, β_*j*_ = fixed effect of litter size (*j* = 12, 13, …, 21), α_*i*_β_*j*_ = interaction between farrowing duration and litter size, c_*k*_ = random batch effect (*k* = 1, 2, …, 8), d_*l*_ = random parity class effect (*l* = 1, 2, or 3; parity 1: class 1, parity 2, 3, and 4: class 2, and parity >4: class 3), f_*m*_ = random feeding treatment effect (*m* = 1, 2, …, 6), and ε_*ijklmn*_ = residual error term. The sow was considered as the experimental unit. For vitality score, the observer was added to the model as a random effect (*n* = 1, 2, or 3). Preliminary analysis in models 2 and 3 demonstrated a lack of effect of the interaction between litter size and farrowing duration. Consequently, results will be expressed per main effect.

For all models and analyses, differences were considered to be significant at *p* ≤ 0.05 and 0.05 < *p* ≤ 0.10 as a tendency. Data are expressed as LSmeans ± SEM or as regression coefficients (β).

## Results

The average TNB was 17.1 ± 3.4 (mean ± SD) piglets per litter, with 16.1 ± 3.1 live born and 1.0 ± 1.4 stillborn (5.8%). It took sows, on average, 236 ± 121 min (range 65–515 min) to complete farrowing. In total, 818 piglets from 109 litters were blood sampled via the umbilical cord, from which 529 samplings harvested enough blood to do both blood gas analyses and lactate analyses, 172 samplings harvested sufficient blood for blood gas analyses only, and 117 samplings were only used to harvest plasma for lactate analyses.

### Litter Size Class and Farrowing Duration Class Interaction

**Figure 1:**
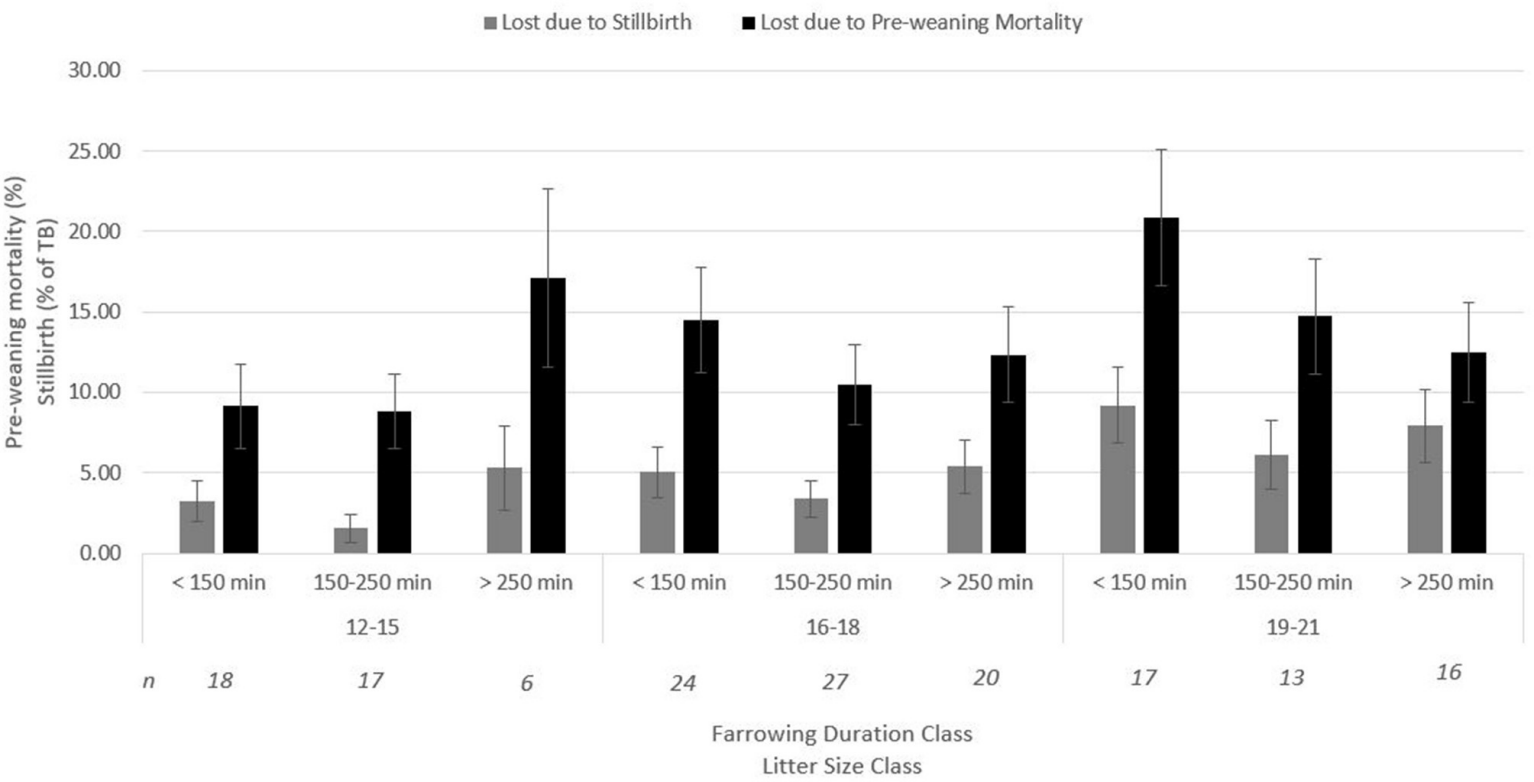
Interaction between farrowing duration (classified) and litter size (classified) on percentage of stillbirth (*p* = 0.77) and pre-weaning mortality (*p* = 0.10) (Lsmeans ± SEM). *n*, number of litters per category.

There was no interaction between litter size class (LSC; small: 12–15 piglets, medium: 16–18 piglets, and large litters: 19–21 piglets) and farrowing duration class (FDC; short: <150 min, medium: 150–250 min, and long: >250 min) on umbilical cord blood gasses, vitality score (data not shown), and percentage of stillborn piglets (Figure 1). A tendency (*p* = 0.10, Figure 1) for an interaction between LSC and FDC was found on the percentage of pre-weaning mortality. For small litters, a long duration of farrowing tended to increase pre-weaning mortality compared with a short and medium farrowing duration, while for large litters, a medium to long duration of farrowing tended to decrease pre-weaning mortality. For medium litter sizes, duration of farrowing did not affect incidence of pre-weaning mortality.

Because no other interactions were found between LSC and FDC, the main effects are presented separately (Tables 1, 2). Table 1 shows the main effects of the level of hypoxia and piglet characteristics per LSC. Large litters showed a significantly higher lactate level (*p* < 0.01), lower average vitality score (*p* = 0.01), and a higher percentage of stillborn piglets (*p* < 0.01) compared with medium and small litters. Other umbilical cord blood gasses were not different between litter size classes. Main effects of FDC on the level of hypoxia as well as piglet vitality, stillbirth, and pre-weaning mortality are shown in Table 2. Piglets born from sows with a short farrowing duration showed a similar partial oxygen pressure in umbilical cord blood compared with piglets born from sows with a long duration of farrowing, but a higher level than piglets born from sows with a medium duration of farrowing. In addition, piglets born from sows with a short duration of farrowing tended to show or showed the lowest acid–base balance (BE_ecf_, *p* = 0.06) and bicarbonate concentration (HCO_3_, *p* = 0.02) in umbilical cord blood compared with piglets born when farrowing duration was medium or long. Oxygen saturation level (sO_2_) tended to be higher (*p* = 0.07) in piglets born from sows with a short duration of farrowing compared with a medium or long duration of farrowing. Vitality score was not different between farrowing duration classes, but percentage of stillborn piglets tended to be higher (*p* = 0.06) in piglets born from sows with a medium duration of farrowing than in piglets born from sows in both other farrowing duration classes.

**Table 1:** Effects of litter size class (total born) on umbilical cord blood parameters immediately after birth, vitality of piglets, incidence of stillbirth, and pre-weaning mortality (LSmeans ± SEM).

	**Litter size class**			**SEM**	***p*-value**
	**12–15**	**16–18**	**19–21**		
*N* (litters)	41	71	47		
Average parity	2.8	3.3	4.4		
**Umbilical cord blood parameters**					
pH	7.52	7.51	7.51	0.02	0.79
*p*CO_2_ (mmHg)	34.9	35.3	34.5	1.32	0.75
*p*O_2_ (mmHg)	35.1	35.6	35.0	1.18	0.90
BEecf (mmol/L)	4.73	4.43	3.78	0.69	0.49
HCO_3_ (mmol/L)	27.5	27.1	26.7	0.61	0.58
sO_2_ (%)	67.8	68.8	66.8	2.09	0.58
Lactate (mmol/L)^1^	3.87^b^	4.12^b^	4.89^a^	0.32	<0.01
Average vitality score^2^	2.29^a^	2.16^ab^	2.09^b^	0.07	0.01
Stillbirth (% of total born)	2.98^b^	4.52^b^	7.65^a^	1.83	<0.01
Pre-weaning mortality (%)	11.2	12.3	15.7	3.10	0.07

1 *Parameters were normalized by using a base 10 logarithm transformation. LSmeans were backtransformed to original scale. Pooled SEM is still reported as transformed value*.

2 *Individual vitality of newborn piglets was scored during the first 30 s after birth by using the scoring method as described by Baxter et al. (16)*.

ab *Different superscripts represent a significant difference between litter size classes*.

**Table 2:** Effects of farrowing duration class on umbilical cord blood parameters immediately after birth, vitality of piglets, incidence of stillbirth, and preweaning mortality (LSmeans ± SEM).

	**Farrowing duration class (min)**			**SEM**	***p*-Value**
	**<150**	**150–250**	**>250**		
*N* (litters)	59	57	43		
Average parity	3.3	3.2	4.0		
**Umbilical cord blood parameters**					
pH	7.51	7.52	7.51	0.02	0.57
*p*CO_2_ (mmHg)	34.4	35.4	34.9	1.38	0.63
*p*O_2_ (mmHg)	37.2^a^	33.8^b^	34.7^ab^	1.23	0.05
BE_ecf_ (mmol/L)	3.23	4.67	5.04	0.71	0.06
HCO_3_ (mmol/L)	26.1^b^	27.6^a^	27.7^a^	0.65	0.02
sO_2_ (%)	70.2	65.8	67.4	2.23	0.07
Lactate (mmol/L)^1^	4.18	4.33	4.37	0.32	0.74
Average vitality score	2.14	2.16	2.23	0.06	0.37
Stillbirth (% of total born)	5.35	3.19	6.10	1.66	0.06
Preweaning mortality (%)	14.2	11.1	13.8	2.94	0.14

1 *Parameters were normalized by using a base 10 logarithm transformation. LSmeans were back transformed to original scale. Pooled SEM is still reported as transformed value*.

2 *Individual vitality of newborn piglets was scored during the first 30 s after birth by using the scoring method as described by Baxter et al. (16)*.

ab *Different superscripts represent a difference (p ≤ 0.05) between farrowing duration classes*.

### Linear Effects of Litter Size

**Figure 2:**
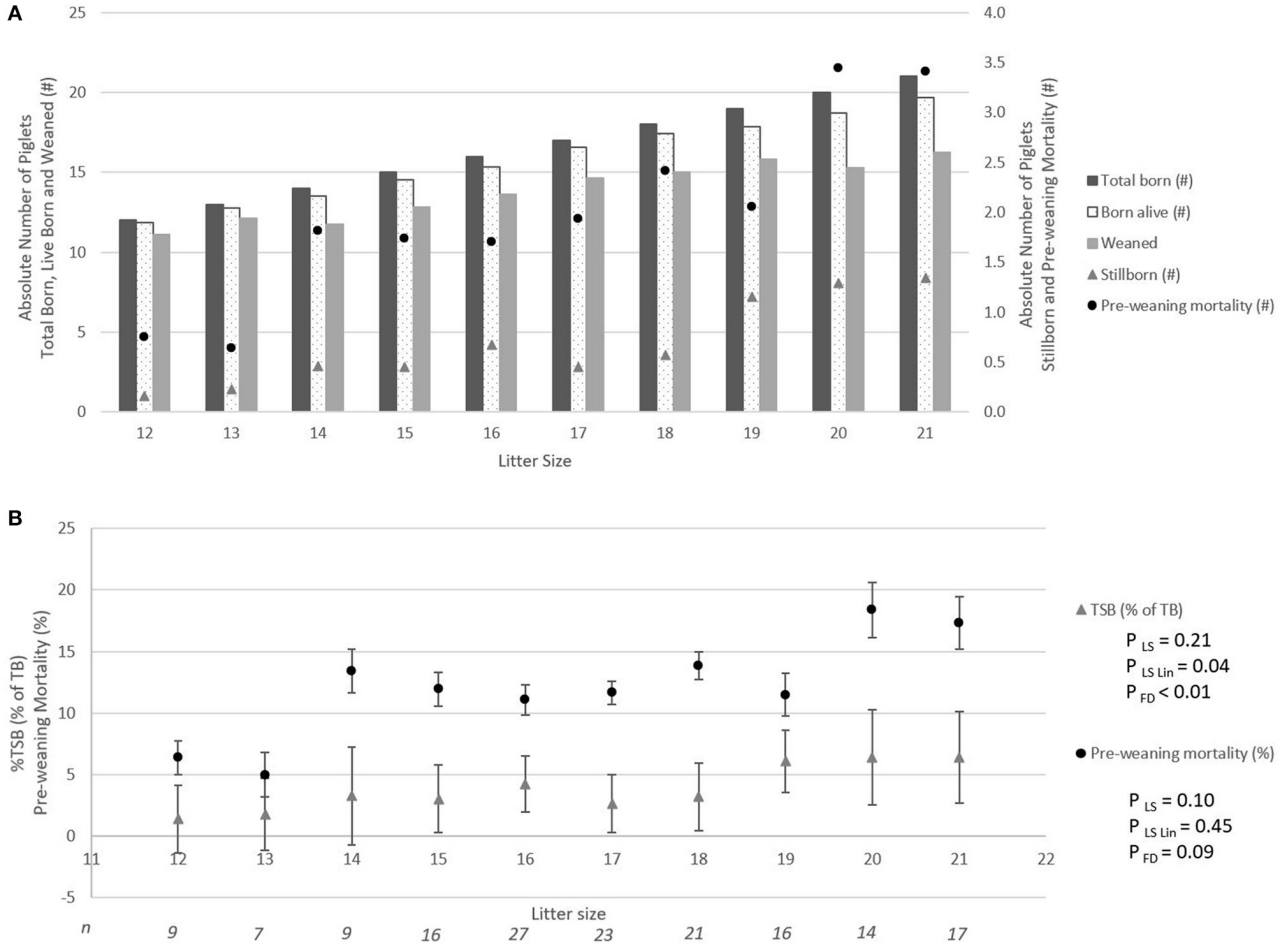
The absolute number of piglets born in total, born alive, stillborn, lost due to pre-weaning mortality, and weaned per litter size **(A)**, and incidence of stillbirth (▴) and pre-weaning mortality (•) per litter size (LSmeans ± SEM) **(B)**. Analyses performed on individual litters. *p*_LS_ is the *p*-value for the fixed effect of litter size. *p*_LSLin_ is the *p*-value for the linear effect of litter size, and *p*_FD_ is the effect of farrowing duration class. N, number of litters per litter size. ^#^Absolute number.

**Figure 3:**
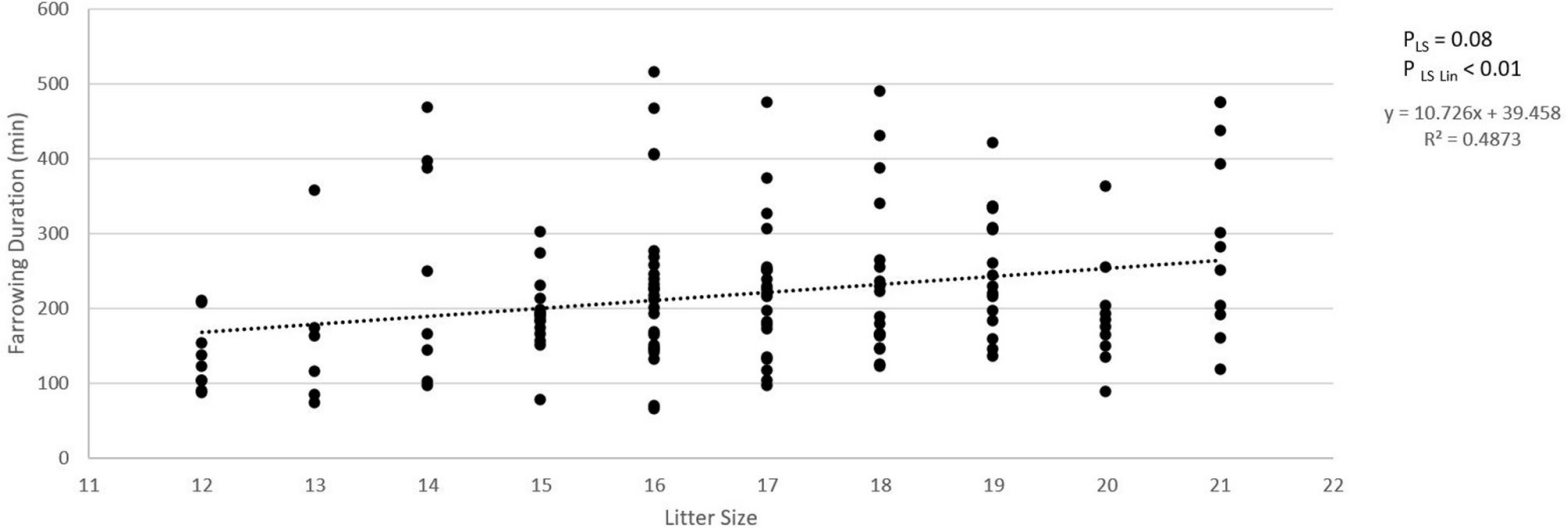
Relationship between litter size and farrowing duration. Total duration of farrowing was defined as the time between the birth of the first and the last piglet in a litter. *p*_LS_ is the *p*-value for the fixed effect of litter size. *p*_LSLin_ is the *p*-value for the linear effect of litter size.

**Figure 4:**
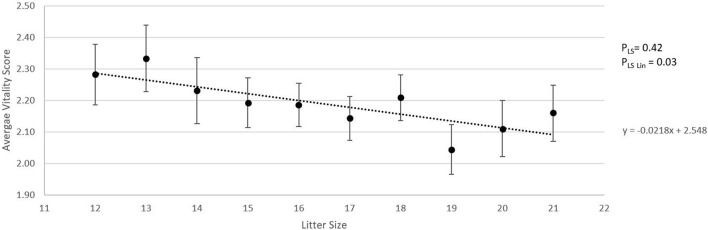
Relationship between litter size and average piglet vitality score conducted within 30 s after birth, using the scoring method as described by Baxter et al. (16) (LSmeans ± SEM). Analysis was performed on individual litters. *p*_LS_ is the *p*-value for the fixed effect of litter size. *p*_LSLin_ is the *p*-value for the linear effect of litter size.

Figure 2A shows the absolute number of piglets born in total, born alive, stillborn, died before weaning, and number of piglets weaned when litter size increased from 12 to 21 piglets. For each additional piglet born to the litter, the percentage of stillbirth increased linearly by 0.5% (*p*_Lin_ = 0.04, Figure 2B). Pre-weaning mortality increased by 1.1% for each additional piglet born to the litter (*p*_Lin_ < 0.01, Figure 1B). Average birth weight of piglets decreased by 17.6 g per piglet with each additional piglet born to the litter (*p*_Lin_ < 0.01). The variation in birth weight within a litter, expressed as SD, was not related to litter size. Farrowing duration increased linearly with litter size (10.7 min per extra piglet between 12 and 21 piglets, Figure 3, *p*_Lin_ < 0.01). Average vitality of piglets within a litter decreased linearly (Figure 4, *p*_Lin_ = 0.03) by 0.02 per piglet when litter size increased from 12 to 21 piglets.

### Linear Effect of Farrowing Duration

**Figure 5:**
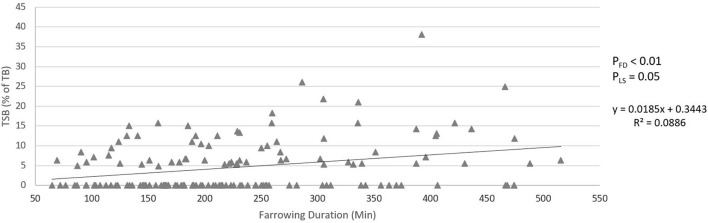
Relationship between farrowing duration and incidence of stillbirth (total still born as percentage of total born). *p*_FD_ is the *p*-value for the effect of farrowing duration. *p*_LS_ is the *p*-value for the fixed effect of litter size.

**Figure 6:**
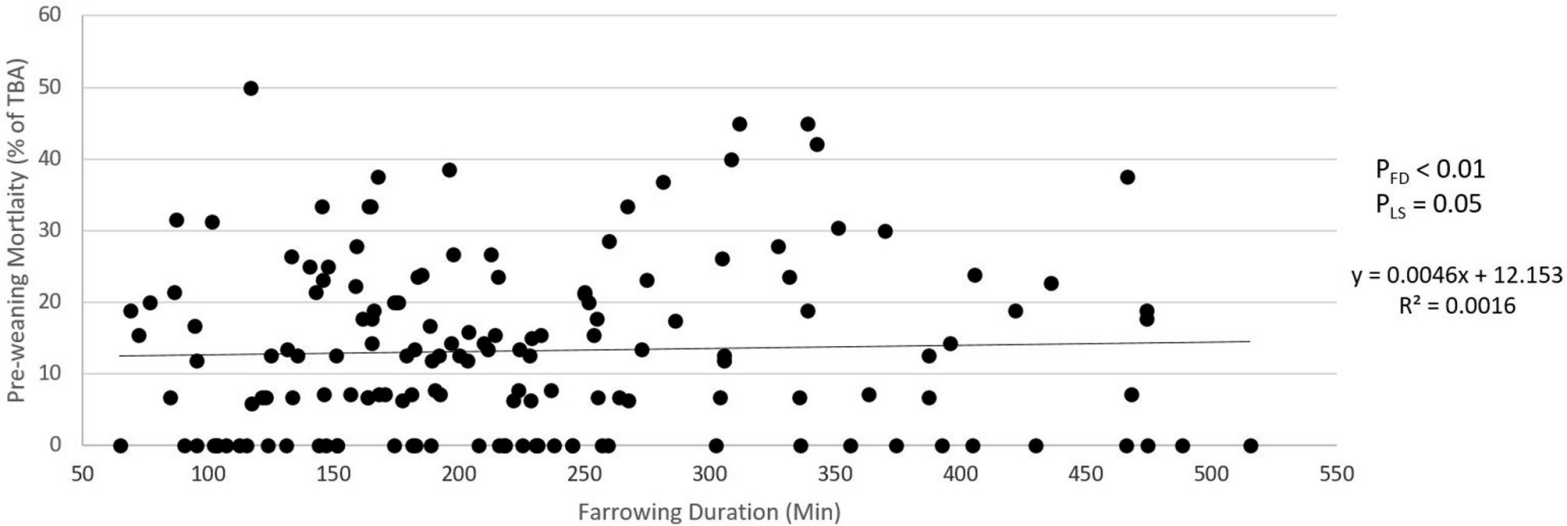
Relationship between farrowing duration and pre-weaning mortality (piglets lost as percentage of total born alive). *p*_FD_ is the *p*-value for the effect of farrowing duration. *p*_LS_ is the *p*-value for the effect of litter size.

Figures 5, 6 show the relationship between farrowing duration and incidence of stillbirth and pre-weaning mortality. Incidence of stillbirth increased by 1.85% per 100 min of farrowing duration (*p*_FD_ < 0.01) and pre-weaning mortality increased by 0.46% per 100 min of farrowing duration (*p*_FD_ < 0.01).

## Discussion

Larger litter sizes are associated with a higher percentage of stillborn piglets (13, 18) and pre-weaning mortality rate (19–21). Larger litter sizes are also associated with longer farrowing duration (5). Because of this entangling of litter size and farrowing duration, it is unclear what the impact of litter size as such is on stillbirth or pre-weaning mortality or what the impact of a prolonged farrowing duration for larger litters is for these losses. This study therefore evaluated effects of litter size, farrowing duration, and their interaction on the level of asphyxia, piglet vitality, and percentage of stillbirth and pre-weaning mortality (excluding stillbirth) within a litter. Litter size class had a strong significant effect (*p* ≤ 0.01) on lactate levels in umbilical cord blood, average vitality score, and stillbirth percentage, all increasing as litter size class got larger. For farrowing duration class, effects were more moderate (*p* ≥ 0.01 or trends), and most favorable levels were mostly observed in piglets born from sows with a medium farrowing duration. This suggests that the effect of litter size on umbilical cord blood gasses, vitality, and stillborn percentage is stronger than the effect of farrowing duration.

Data leveraged in the current study originate from a study evaluating a dose response of maternal nitrate supplementation [as described in (14, 15)] in which no effect of treatment was found on duration of farrowing (14), litter size, and incidence of stillbirth (15). There tended to be a quadratic effect of dosage of nitrate supplementation on pre-weaning mortality; however, all dietary treatments were distributed approximately equally across all farrowing durations.

### Litter Size Class and Farrowing Duration Class Interaction

No interaction was found between LSC and FDC on umbilical cord blood parameters, piglet vitality score, and percentage of stillborn piglets. A tendency for an interaction between LSC and FDC was found on percentage of pre-weaning mortality. Litters of 12–15 piglets showed the lowest incidence of pre-weaning mortality when farrowing duration did not exceed 250 min. In litters of 16–18 piglets, no effect of farrowing duration was found on percentage of pre-weaning mortality, but in litters of 19–21 piglets, it appeared to be beneficial to have a longer farrowing duration, since pre-weaning mortality rate tended to decrease as farrowing duration increased. These results suggest that, looking at pre-weaning mortality, the optimal farrowing duration might differ per LSC. Strikingly, the same interaction was not seen in relation to stillbirth incidence, which was expected to be more directly linked to farrowing duration than pre-weaning mortality. However, it has to be mentioned that cross fostering was applied and therefore pre-weaning mortality numbers included piglets from the birth litter as well as piglets fostered onto a sow. Piglets were not cross fostered solely to other sows within the same farrowing duration group. English and Wilkinson (22) showed that piglets that died before 3 weeks of age had higher blood lactate concentrations at birth than piglets that survived (383.3 vs. 303.0 μg of lactate/ml of blood for piglets that died and survivors, respectively; *p* < 0.01), showing that the level of asphyxiation at birth appears to be related to pre-weaning mortality rate. Asphyxia at birth impacts the time to reach the udder and the quantity of colostrum ingested (23), but also, likely linked to that, asphyxia is linked to lower body temperatures and consequently higher risks of death due to hypothermia (24–26). However, in the current study, vitality scores and umbilical cord acid–base blood parameters did not reflect the effect found on pre-weaning mortality. Although both piglet vitality score (16) as well as umbilical cord acid–base blood parameters at birth (22) have been linked to pre-weaning mortality percentage's other indicators, such as time to reach the udder, colostrum intake, or body temperatures (which were not evaluated in this study), they may have reflected the effect found on pre-weaning mortality.

Average duration of farrowing of sows in the current study was short (average 236 ± 121 min with an average litter size of 17.1 ± 3.4 piglets) compared with other studies. The highest FDC contained sows exceeding 250 min of farrowing time, which is below the formerly indicated threshold level of 300 min, after which the risk for stillbirth increased (in a herd with an average litter size of 12.7 piglets) (11). Level of asphyxia and incidence of stillbirth increases with birth order (22, 27), and average blood *p*CO_2_ increases with litter size (27), which might explain why larger litters show a higher incidence of asphyxia and stillbirth. However, Langendijk et al. (23) reported the risk of being a stillborn for piglets with birth order 13 or up was 9% when farrowing duration took <280 min and 23% when farrowing duration exceeded 280 min. This suggests that the total farrowing duration seems to surpass the effect of birth order. It can be speculated that the main driver for piglet losses is the power of and frequency of uterine contractions rather than just the duration of farrowing. Uterine contractions decrease blood flow of the uterus and gaseous exchange through the placenta (28). A short duration of farrowing with powerful contractions will likely increase intrapartum death, which is also demonstrated in studies evaluating the effect of oxytocin treatment (29, 30). Although it is likely that polytocous species experience an increased number of uterine contractions during parturition compared with monotocous species (31), it is unclear whether or not the power and frequency of contractions during parturition is different between sows with different litter sizes. However, it is known that uterine blood flow per fetus decreases when litter size increases (32), likely caused by a smaller placenta (10, 16). Consequently, it can be speculated that the effect of powerful contractions (as hypothesized to be the case during short duration of farrowing and high oxytocin levels) might be more detrimental in larger litters when piglets are already subjected to a lower blood flow compared with smaller litters. This could explain the trend for a higher incidence of pre-weaning mortality in larger litters with a short duration of farrowing.

### Litter Size

LSC significantly influenced incidence of stillbirth, umbilical cord lactate levels, and average vitality score of the litter. Successive uterine contractions can lead to a repetitive obstructed blood flow to the fetus, causing a more anaerobic metabolism, which is represented in umbilical cord lactate levels (33, 34). Perinatal asphyxia has been related to a lower post-natal vitality and higher post-natal mortality until 10 days of age (27), which might be caused by altered expression patterns of stress-related proteins in the brain, heart, and intestines (35). The significant decrease in average vitality score and the trend for a higher pre-weaning mortality percentage in the higher LSC found in the current study are aligned with this hypothesis. In addition, studies have shown an increase in umbilical cord blood pH as birth order increases (27, 34), which is related to higher average umbilical cord lactate levels of litters in higher litter size classes as measured in the current study. When analyzed linearly, the increase in litter size was, in our study, related to an increase in farrowing duration. Farrowing duration increased by 10.7 min per piglet additionally born to the litter. Combining data on average litter size and farrowing duration of several studies published between 2004 and 2018 (5, 11–13, 36–39) resulted in an estimation of 44 min of extra farrowing time per piglet added to a litter. This is considerably higher than the 11 min in the current study, which might be related to the relatively short duration of farrowing seen in the current study as will be further discussed below.

### Farrowing Duration

As mentioned before, farrowing duration of sows in the current study was relatively short compared with other studies despite of no interventions being used. The sows in our study farrowed, on average, 17.1 ± 3.4 piglets in 236 ± 121 min (13.8 min per piglet). Feyera et al. (40) found a duration of farrowing of 348 ± 162 min for sows with an average litter size of 17.5 ± 3.8 (19.9 min per piglet), and Björkman et al. (36) found a farrowing duration of 396 ± 234 min for 16.3 ± 3.6 piglets (24.3 min per piglet). The difference in farrowing duration among studies might be caused by sow breed, sow body condition, management, parity, piglet birth weight, or feeding regime around farrowing (1, 5, 11, 37, 41–43). Based on this variation among studies, it can be concluded that farrowing duration should always be considered in the perspective of specific farm circumstances. This also suggests that using a fixed threshold for maximum farrowing duration before interventions should take place, as suggested by Langendijk et al. and Oliviero et al. (11, 12), should be considered with care. Several studies found a relationship between farrowing duration and the incidence of stillbirth (11, 13, 18, 23), while the relationship between farrowing duration and pre-weaning mortality is less described (16, 22), but also less clear. This is in line with findings in our study, which showed that farrowing duration did influence incidence of stillbirth. When classified in a short, medium, or long farrowing duration, incidence of stillbirth tended to be lowest when farrowing duration was between 150 and 250 min (medium duration) when comparing a short and long duration of farrowing. This is likely caused by the percentage of sows within each class lacking stillborn piglets, which was considerably higher in sows with a medium duration of farrowing than in both other farrowing duration classes (46.0, 65.1, and 39.5% for a short, medium, or long farrowing duration class, respectively). These results suggest an optimum in farrowing duration in relation to the incidence of stillbirth, instead of shorter being better. On the one hand, duration of farrowing should not be too short, since intense uterine contractions and abdominal straining may reduce placental blood flow and, therefore, the oxygen exchange between mother and fetus (7). On the other hand, a prolonged duration of farrowing can result in a higher risk for hypoxia for the piglets, since piglets are longer subjected to successive uterine contractions and potentially impaired oxygen exchange (6, 27). Umbilical cord blood parameters seem to give a limited picture on the course of parturition and did not align well with the stillborn rate in the current study. Blood pH, *p*CO_2_, and lactate were not significantly affected by farrowing duration class, whereas *p*O_2_ and HCO_3_ was affected, and BE_ecf_ tended to be affected. Most favorable levels (high *p*O_2_, low BE_ecf_, and low HCO_3_) were observed in piglets born from sows with a short farrowing duration (<150 min), and levels were not significantly different between piglets born from sows with a medium (150–250 min) or long (>250 min) duration of farrowing. Findings are partly in line with those of van Dijk et al. (44), who divided parturition in three parts and found significantly lower umbilical cord blood pH, HCO_3_, and BE_ecf_, and significantly higher *p*CO_2_ in piglets born in the last third part compared with piglets born in the first and second third part of parturition. Based on the current results, it can be hypothesized that optimal farrowing duration is not fixed, but depends on litter size, as discussed above.

## Conclusion

This study provides further evidence to support the influence of high litter sizes and long farrowing durations on the incidence of stillbirth and pre-weaning mortality. Of these two factors studied, litter size is suggested to be a larger driver for stillbirth than farrowing duration, since a clear linear relationship was found between litter size and incidence of stillbirth. For pre-weaning mortality, litter size and farrowing duration tended to interact, suggesting that optimal farrowing duration depends on litter size. Setting a fixed threshold for maximum farrowing duration to intervene in the farrowing process should, thus, be handled with care.

## Data Availability Statement

The raw data supporting the conclusions of this article will be made available by the authors, without undue reservation.

## Ethics Statement

The animal study was reviewed and approved by Animal Use and Care Committee of Wageningen University, Netherlands, in accordance with EU Directive 2010/63/EU for animal experiments.

## Author Contributions

MB was responsible for the study design, planning and setting up of the experiment, data collection, data analysis and interpretation, and was the principal author of the manuscript. IL contributed in the experimental design, data analysis, and result interpretation. BK reviewed and advised on the study design, supported the data interpretation, and commented on the manuscript. HB supported and advised on the study design, planning and setting up the experiment, data collection, data interpretation, and manuscript review. All authors contributed to the article and approved the submitted version.

## Funding

This article has been derived from a research project funded by the Cargill Animal Nutrition. The funder was not involved in the study design, collection, analysis, interpretation of data, the writing of this article or the decision to submit it for publication.

## Conflict of Interest

MB and IL were employed by Cargill b.v. The remaining authors declare that the research was conducted in the absence of any commercial or financial relationships that could be construed as a potential conflict of interest.

## Publisher's Note

All claims expressed in this article are solely those of the authors and do not necessarily represent those of their affiliated organizations, or those of the publisher, the editors and the reviewers. Any product that may be evaluated in this article, or claim that may be made by its manufacturer, is not guaranteed or endorsed by the publisher.
